# Supplementary material for: Censcyt: censored covariates in differential abundance analysis in cytometry
Source: BMC Bioinformatics. 2021 May 10;22:235. doi: 10.1186/s12859-021-04125-4 (PMC8108359; doi:10.1186/s12859-021-04125-4)
Supplement: Supplementary file 1 — Additional file 1. Supplementary figures. [file 12859_2021_4125_MOESM1_ESM.pdf]

Supplementary Figures

censcyt: censored covariates in differential abundance analysis in cytometry

April 10, 2021

Reto Gerber, Mark D. Robinson

Department of Molecular Life Sciences, University of Zurich, Switzerland
SIB Swiss Institute of Bioinformatics, Switzerland

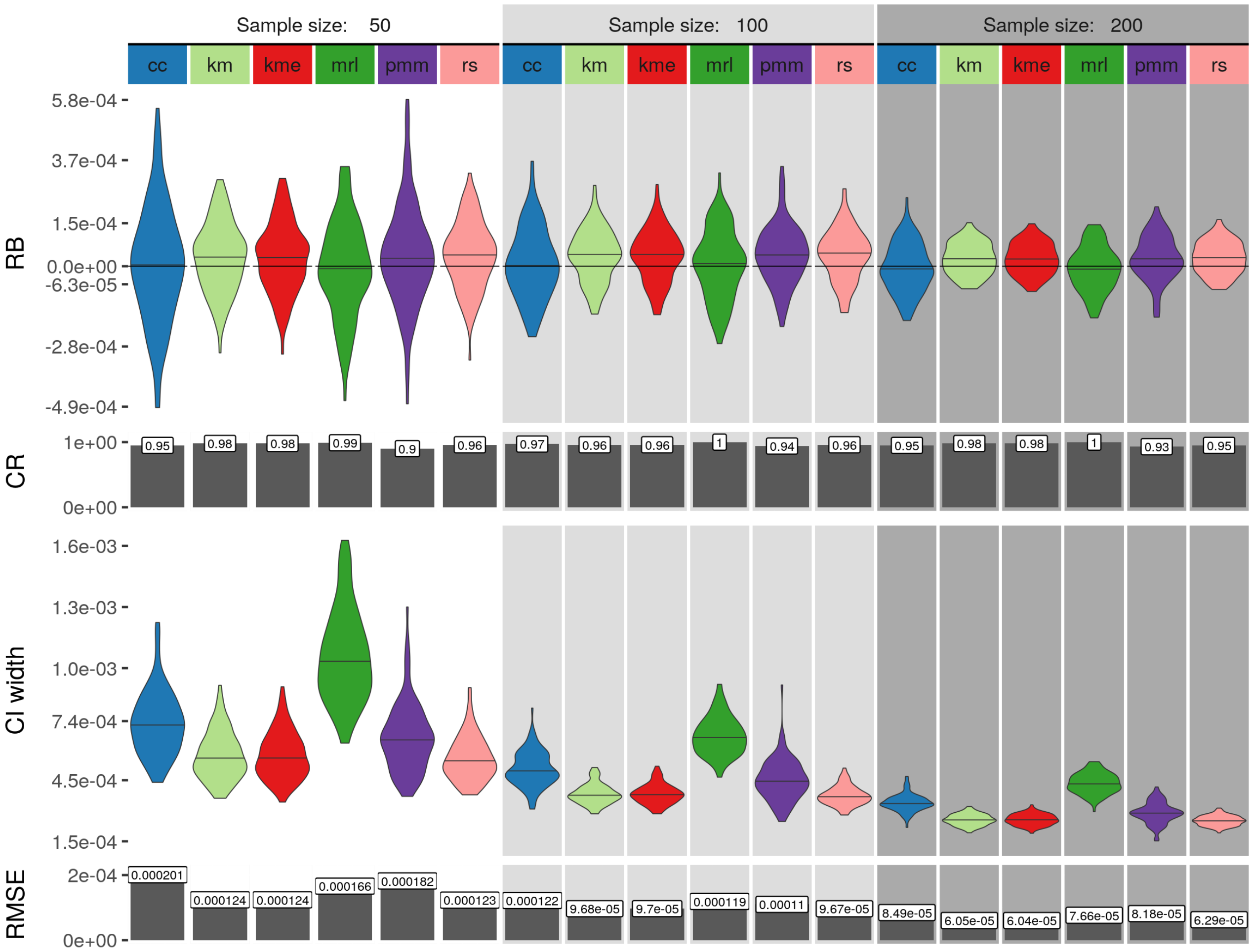

Figure S1: Single cluster simulation results for a censoring rate of 50% for sample sizes of 50, 100 and 200. Shown are four measures calculated from 100 simulation repetitions: raw bias (RB), coverage rate (CR), confidence interval (CI) width and root mean squared error (RMSE). *cc*: complete case analysis, *km*: Kaplan-Meier imputation, *kme*: Kaplan-Meier imputation with an exponential tail, *mrl*: mean residual life imputation (conditional multiple imputation), *pmm*: predictive mean matching (treating censored values as missing), *rs*: risk set imputation. Other parameter values are: true regression coefficient $\beta_1 = -1e - 4$, number of multiple imputations = 50 and the variance of the random effect = 1.

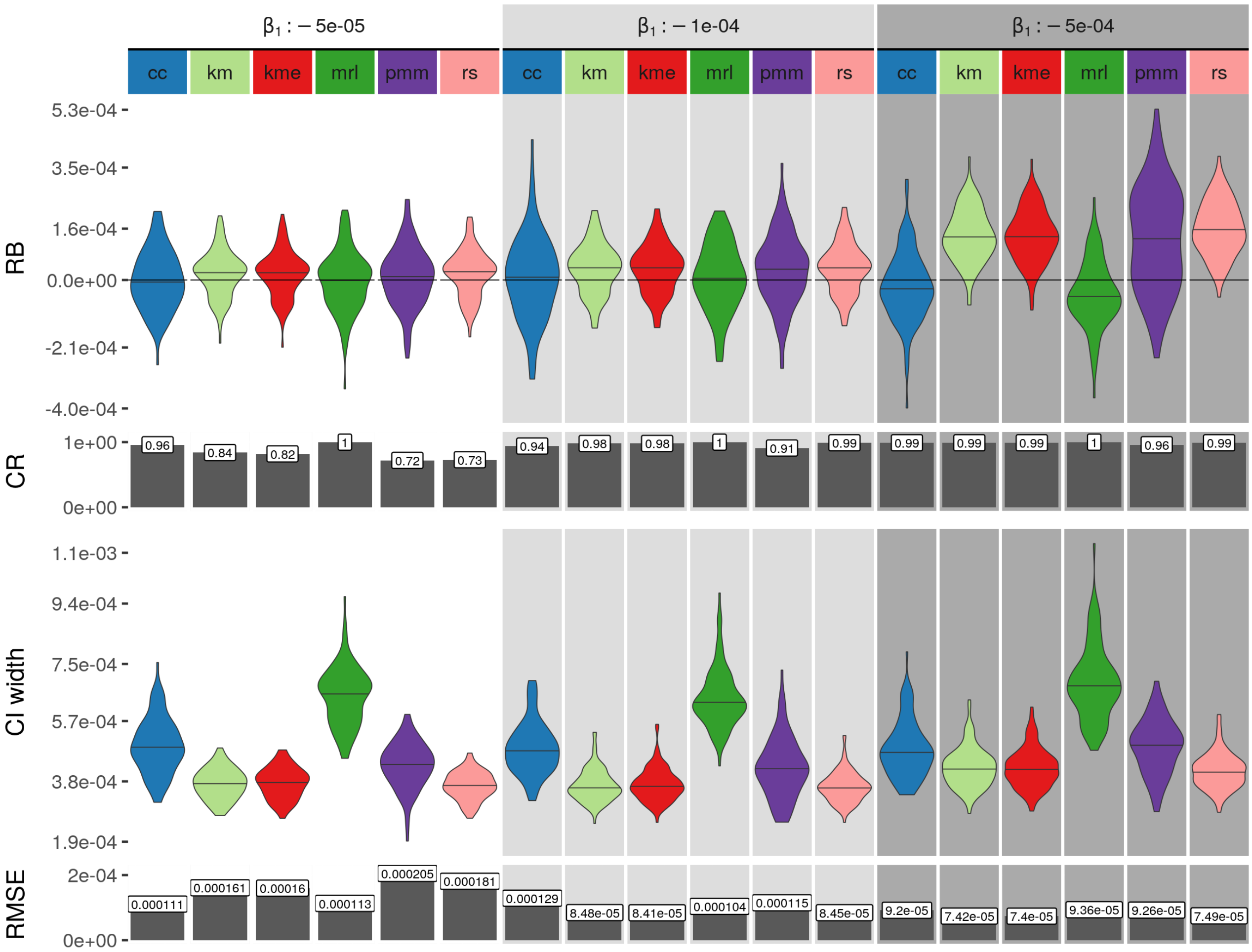

Figure S2: Single cluster simulation results for a sample size of 100 and a censoring rate of 50% for regression coefficients β_1 of $-5e-5$,$-1e-4$ and $-5e-4$. Shown are four measures calculated from 100 simulation repetitions: raw bias (RB), coverage rate (CR), confidence interval (CI) width and root mean squared error (RMSE). *cc*: complete case analysis, *km*: Kaplan-Meier imputation, *kme*: Kaplan-Meier imputation with an exponential tail, *mrl*: mean residual life imputation (conditional multiple imputation), *pmm*: predictive mean matching (treating censored values as missing), *rs*: risk set imputation. Other parameter values are: number of multiple imputations = 50 and the variance of the random effect = 1.

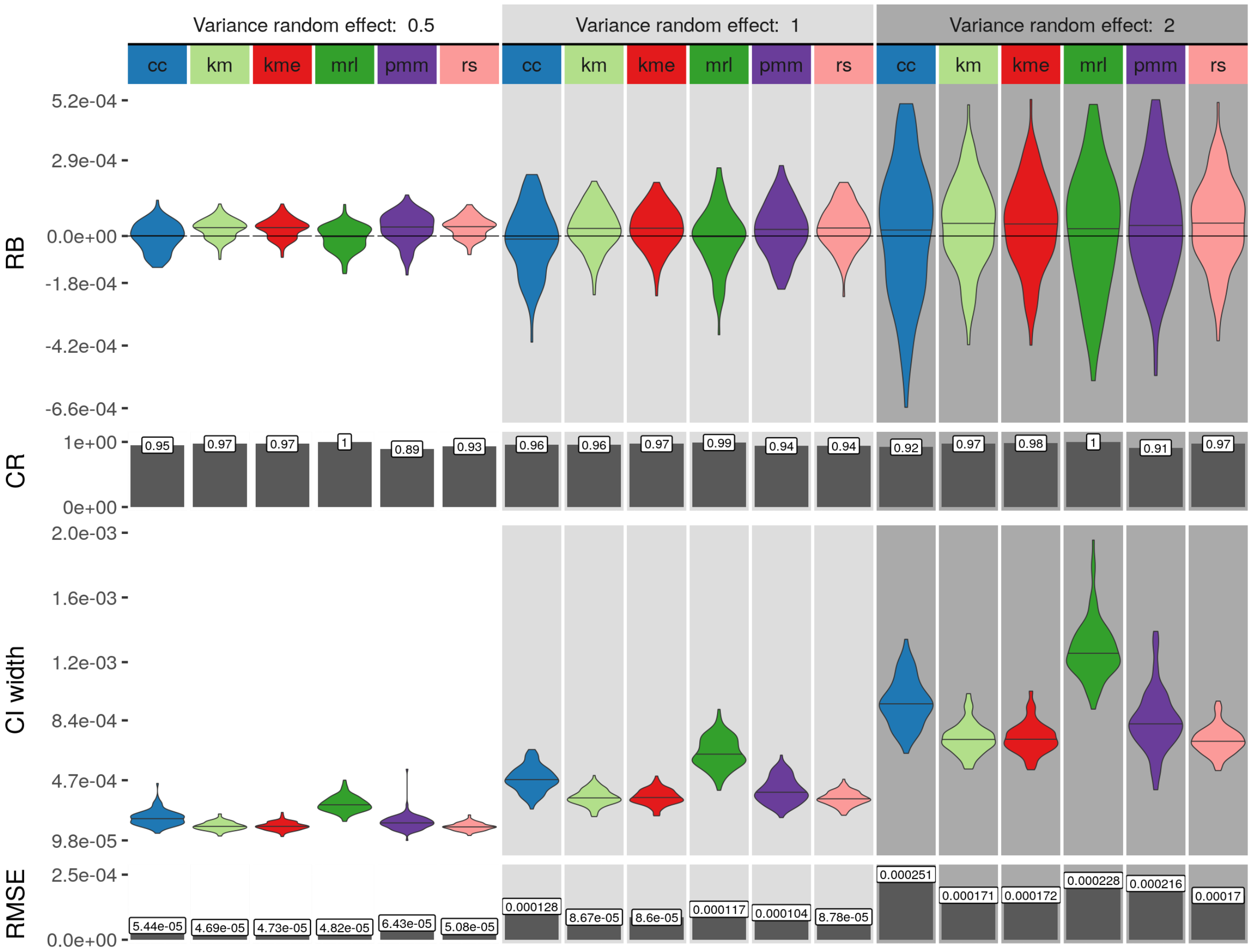

Figure S3: Single cluster simulation results for a sample size of 100 and a censoring rate of 50% for variances of the random effect of 0.5, 1 and 2. Shown are four measures calculated from 100 simulation repetitions: raw bias (RB), coverage rate (CR), confidence interval (CI) width and root mean squared error (RMSE). *cc*: complete case analysis, *km*: Kaplan-Meier imputation, *kme*: Kaplan-Meier imputation with an exponential tail, *mrl*: mean residual life imputation (conditional multiple imputation), *pmm*: predictive mean matching (treating censored values as missing), *rs*: risk set imputation. Other parameter values are: true regression coefficient $\beta_1 = -1e - 4$ and number of multiple imputations = 50.

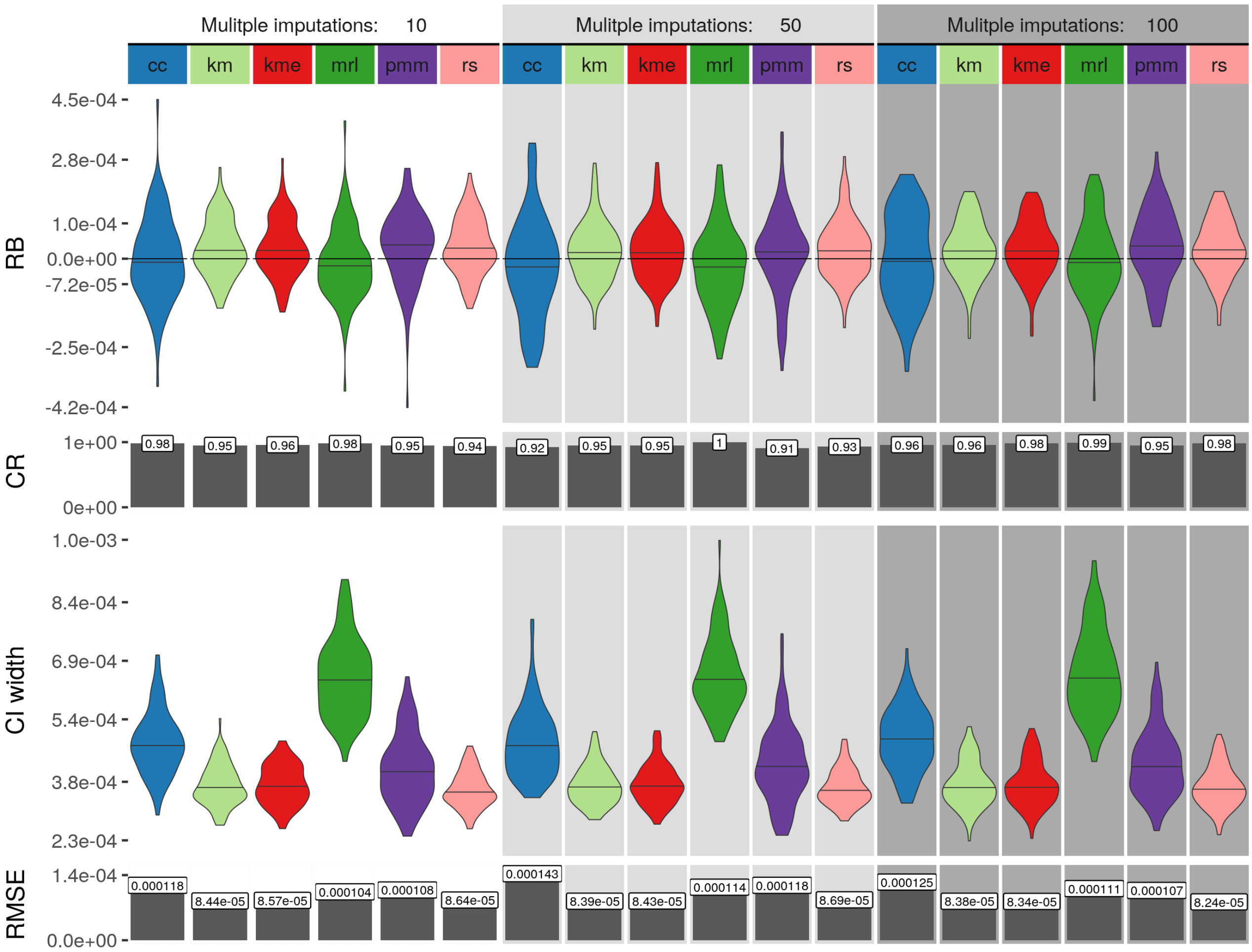

Figure S4: Single cluster simulation results for a sample size of 100 and a censoring rate of 50% for number of imputations of 10, 50 and 100. Shown are four measures calculated from 100 simulation repetitions: raw bias (RB), coverage rate (CR), confidence interval (CI) width and root mean squared error (RMSE). *cc*: complete case analysis, *km*: Kaplan-Meier imputation, *kme*: Kaplan-Meier imputation with an exponential tail, *mrl*: mean residual life imputation (conditional multiple imputation), *pmm*: predictive mean matching (treating censored values as missing), *rs*: risk set imputation. Other parameter values are: true regression coefficient $\beta_1 = -1e - 4$ and the variance of the random effect $= 1$.

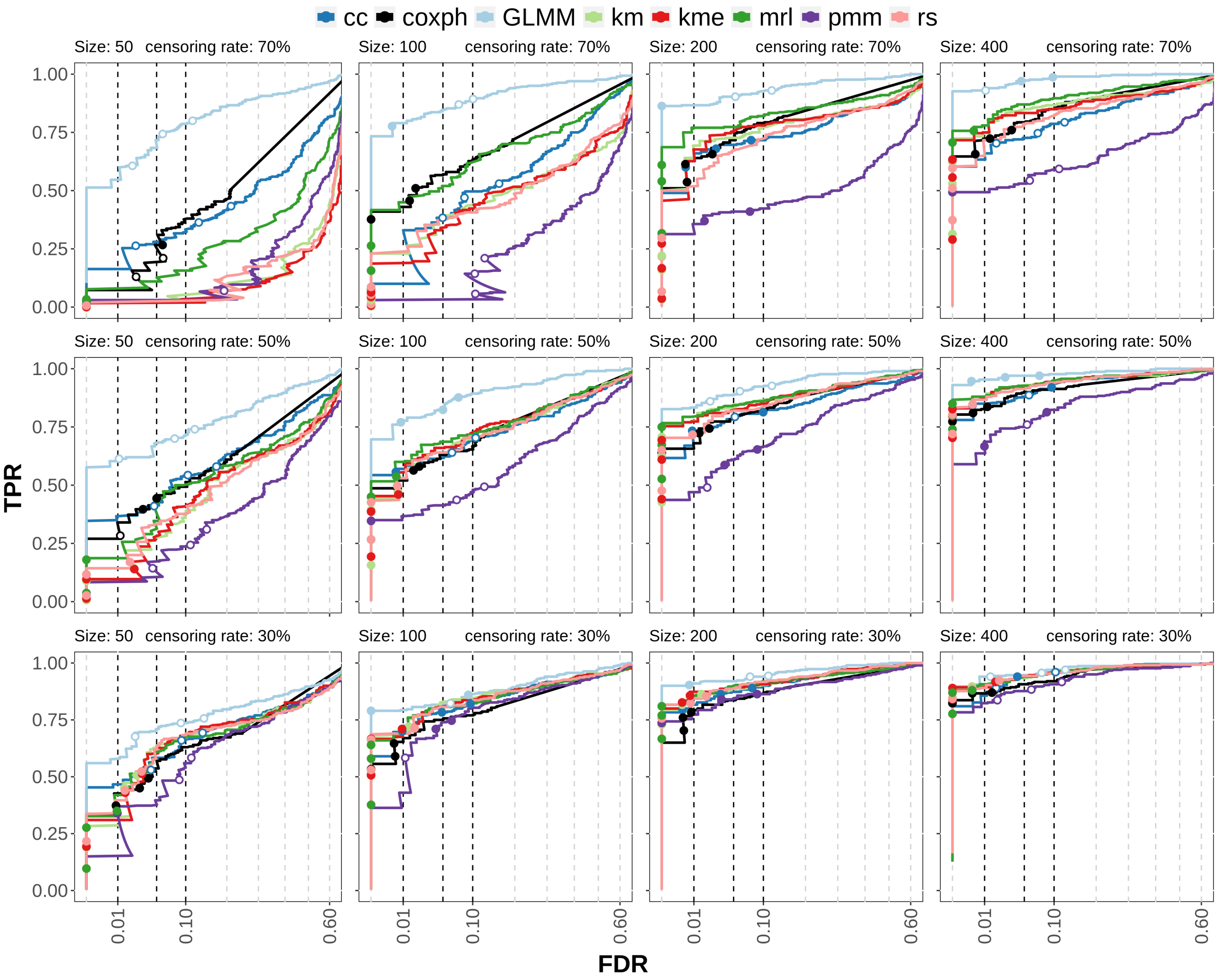

Figure S5: Multiple cluster simulation results testing with only a single (censored) covariate. True positive rate (TPR) vs. False discovery rate (FDR) curves for censoring rates of 30%, 50% and 70% (rows) and samples sizes of 50, 100, 200, 400 (columns). Dots represent values at different significance thresholds (0.01, 0.05, 0.1; dashed lines). Filled dots have a lower FDR than the corresponding threshold, while empty dots have a FDR above. The x-axis is square root transformed. *cc*: complete case analysis, *km*: Kaplan-Meier imputation, *kme*: Kaplan-Meier imputation with an exponential tail, *mrl*: mean residual life imputation (conditional multiple imputation), *pmm*: predictive mean matching (treating censored values as missing), *rs*: risk set imputation, *coxph*: Cox proportional hazards model. *GLMM* uses the (unobserved) ground truth of the survival time and can be considered to be the maximum possible performance of the other methods.

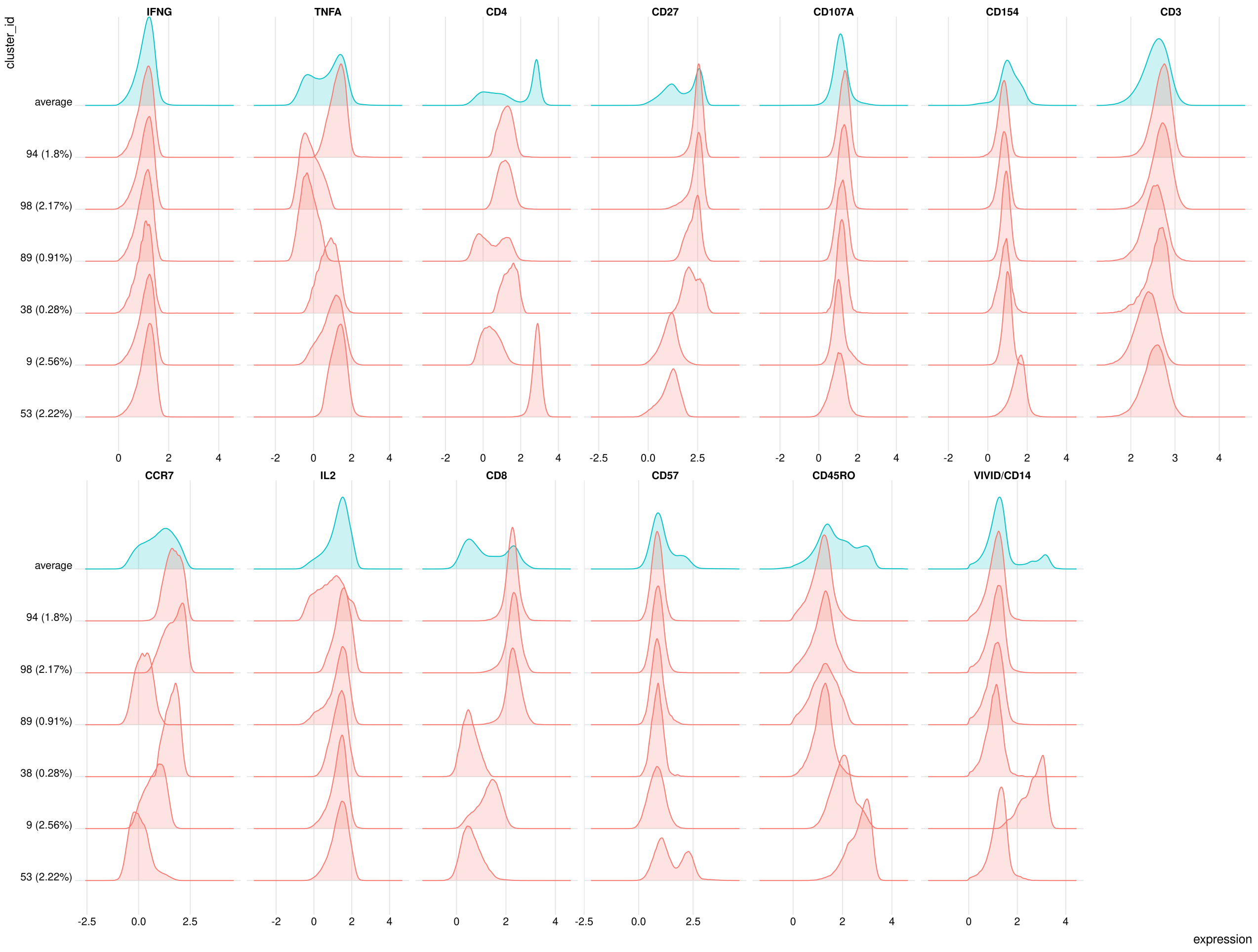

Figure S6: Expressions of top subpopulations of the clustering at a resolution of 100 clusters in the case study. The top row ("average") is the average over all 100 clusters.
